# Human umbilical cord mesenchymal stem cells-derived exosomes for treating traumatic pancreatitis in rats

**DOI:** 10.1186/s13287-022-02893-1

**Published:** 2022-05-26

**Authors:** Li Han, Zhirong Zhao, Xingyun Chen, Ke Yang, Zhen Tan, Zhu Huang, Lichen Zhou, Ruiwu Dai

**Affiliations:** 1 General Surgery Center, General Hospital of Western Theater Command, No. 270, Rongdu Rd, Jinniu District, Chengdu, 610083 Sichuan Province China; 2 College of Medicine, Southwest Jiaotong University, Chengdu, 610031 China; 3 Department of Hepatobiliary and Pancreatic Surgery, Integrated Traditional Chinese and Western Medicine Hospital of Liangshan Yi Autonomous Prefecture, Xichang, 615000 Sichuan Province China; 4 Department of Cardiovascular Surgery, General Hospital of Western Theater Command, Chengdu, 610083 Sichuan Province China; 5 College of Clinical Medicine, Southwest Medical University, Luzhou, 646000 Sichuan Province China

**Keywords:** Exosomes, Human umbilical cord mesenchymal stem cells, Traumatic pancreatitis, Apoptosis, Inflammation, Therapeutic effect

## Abstract

**Background:**

The therapeutic and protective effects of human umbilical cord mesenchymal stem cells-exosomes (hucMSC-Exs) on traumatic pancreatitis (TP) remain unknown. Here, we established a rat model of TP and evaluated and compared the therapeutic effects of hUC-MSCs and hucMSC-Exs.

**Methods:**

HucMSC-Exs were obtained by ultracentrifugation and identified using transmission electron microscopy and western blot analysis. TP rats were treated by tail vein injection of hUC-MSCs and hucMSC-Exs. Their homing in rats was observed by performing fluorescence microscopy. The degree of pancreatic tissue damage was assessed by HE staining, the expression levels of amylase, lipase, and inflammatory cytokines were detected by ELISA, apoptosis was detected by TUNEL assay, and the expression levels of various apoptosis-related proteins were detected by western-blot. The expression levels of apoptosis-related molecular markers were detected by RT-qPCR.

**Results:**

The colonization of exosomes was observed in pancreatic tissue. Compared to TP group, the histopathological score of pancreas was significantly decreased in the TP + hUC-MSCs group and TP + hucMSC-Exs group (*P* < 0.05). Compared to TP group, the activity of serum amylase and lipase was significantly decreased (*P* < 0.05). The expression levels of IL-6 and TNF-α were significantly decreased, while those of IL-10 and TGF-β were significantly increased (*P* < 0.05). The apoptosis index of the TP group was significantly increased (*P* < 0.05), whereas that of the TP + hUC-MSCs and TP + hucMSC-Exs groups was significantly decreased (*P* < 0.05). Compared to TP group, the expression levels of Bax, Bcl-2, and Caspase-3 were significantly decreased in the TP + hUC-MSCs group and TP + hucMSC-Exs group (*P* < 0.05).

**Conclusion:**

HucMSC-Exs can colonize injured pancreatic tissue, inhibit the apoptosis of acinar cells, and control the systemic inflammatory response to facilitate the repair of pancreatic tissue.

## Background

Traumatic pancreatitis (TP) is an acute, non-infectious, inflammation of pancreatic tissues caused by crushing forces that compress the pancreas against the vertebral column. Although the incidence of TP is less than 2% of all blunt force abdominal injuries, its mortality and morbidity can reach as high as 34–60%, with the majority of the mortality being secondary to or concomitant with other organ injuries [1]. After pancreatic injury, the massive spillage of pancreatic juice can directly corrode and irritate the peritoneum, abdominal organs, and blood vessels, which can lead to severe complications [2]. Compared with the traumatic injuries to the liver, spleen, and kidneys, TP is relatively difficult to diagnose in the early stage, as the pancreas is located behind abdominal viscera surrounded by multiple organs, and cannot be easily located after injury [3, 4]. In addition, it remains unknown whether the treatment of TP is affected by its etiology. Current clinical management strategies provide symptomatic and supportive treatment at the late stage. So far, no effective treatment strategy has been proposed to control acinar cell apoptosis and inhibit inflammatory response after pancreatic injury. Therefore, it is crucial to discover an ideal therapeutic measure that can control the progression of the inflammatory response and inhibit apoptosis of acinar cells after pancreatic injury at the early stage to promote the repair of injured pancreatic tissue.

Human umbilical cord mesenchymal stem cells (hUC-MSCs) have been widely evaluated in the field of tissue engineering owing to their ability of self-renewal and multilineage differentiation [5]. In addition, hUC-MSCs surface antigens are not prominently immunogenic, and thus, their rejection was relatively minor after transplantation [6–8]. Thus, it is believed that the hUC-MSCs may play a key role in tissue repair by releasing exosomes (hucMSC-Exs) that directly act on target cells. HucMSC-Exs have been applied in tissue repair, controlling inflammation, and treatment of ischemic diseases as they carry biologically active molecules such as nucleic acids, proteins, and lipids [9–12].

In this study, we established a rat model of TP to explore the safety and efficacy of hucMSC-Exs transplantation for the treatment of TP. We believe that our study outcomes provide a theoretical basis and a new direction for clinical treatment of TP in the future.

## Methods

### Experimental materials

Sprague Dawley (SD) rats, weighing 200–250 g, were purchased from Chengdu Dashuo Experimental Animal Co., Ltd. (animal license No.: SCXK (Chuan) 2020-030). The multifunctional animal impact equipment has been authorized patent (self-developed, patent number: ZL 2016 1 0347341.5). hUC-MSCs were provided by the Chengdu KangErmei Biological Cell Preparation Center (Number: G01210001). The ELISA kits for serum amylase, lipase, and rat IL-6, IL-10, TGF-β, and TNF-α were purchased from Shanghai Jiaocai Biological Technology Co., Ltd. Bax, Bcl-2, and caspase-3, and β-tubulin antibodies were purchased from Affinity Biosciences (Cincinnati, USA). TUNEL kits was purchased from Roche Group (Switzerland).

## Methods

### Extraction and identification of hucMSC-Exs

hUC-MSCs were removed from liquid nitrogen and thawed rapidly. Then, they were cultured in DMEM/F12 at 37 °C in a 5% CO_2_ incubator. Subculture was performed when the cell growth density reached 90%. Cells from passages 3–5 were selected for subsequent experiments. Surface-specific antigens of hUC-MSCs, including CD29, CD44, CD45, and CD34, were identified using flow cytometry. hUC-MSCs were centrifuged at 2000×*g* and 4 °C for 30 min, followed by centrifugation at 10,000 × *g* and 4 °C for 45 min again. The supernatant was collected, filtered through a 0.45 μm filter membrane, transferred to a new centrifuge tube, and centrifuged at 100,000 × *g* and 4 °C for 70 min. The obtained supernatant was discarded. Then, ultracentrifugation was performed at 4 °C and 100,000 × *g* for 70 min. The supernatant was discarded, and the precipitate was resuspended in 200 μL phosphate-buffered saline (PBS). The isolated partial hucMSC-Exs were observed using a transmission electron microscope (Hitachi, HT-7700) and assessed with western blotting. Additionally, the size of hucMSC-Exs was measured using a nanoparticle size analyzer (PARTICLE METRIX, ZetaVIEW). The remaining exosomes were stored at − 80 °C.

### Experimental grouping and establishment of an animal model

**Fig. 1:**
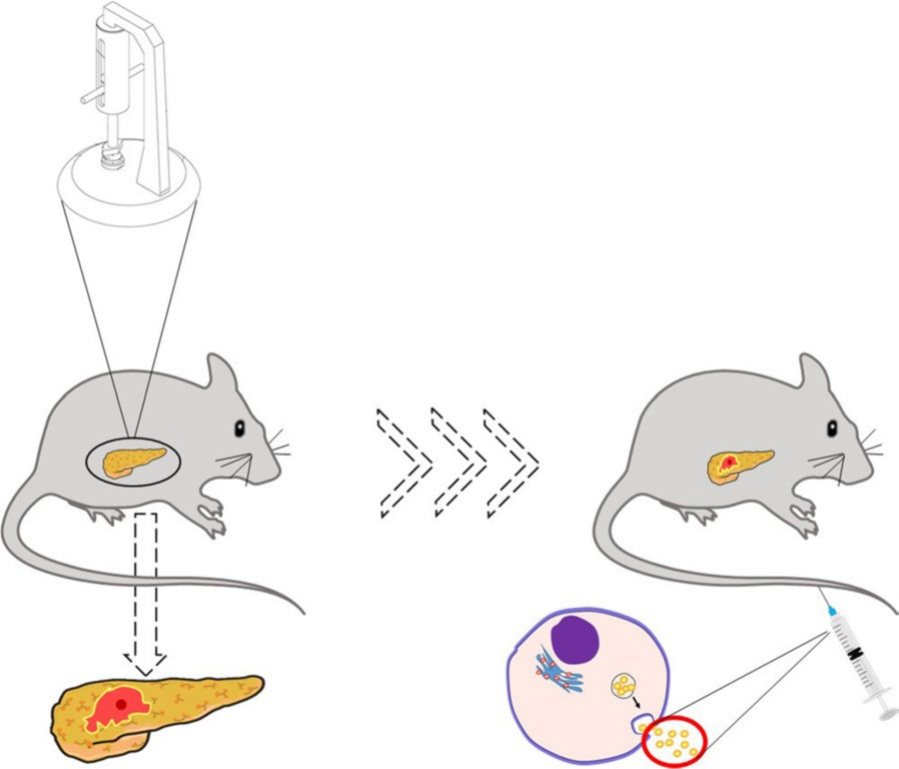
After traumatic pancreatitis modeling, rats were injected with hucMSC-Exs via the tail veins

Forty SD rats were randomly divided into four group, namely the control group (Control group), TP group, human umbilical cord mesenchymal stem cells group (TP + hUC-MSCs group), and human umbilical cord mesenchymal stem cells-Exosomes group (TP + hucMSC-Exs group). In the control group, rats were fixed after anesthesia and laparotomy was performed. The pancreas was gently turned over several times with sterile cotton swabs and then closed. Approximately 1 ml normal saline was injected via the tail veins of rats. In the TP group, rats were anesthetized and fixed as mentioned above and pancreatic tissues were gently isolated. An animal model of TP was established via controlling the injury area using a self-developed multifunctional impactor [13]. Pancreatic tissue was percussed and squeezed at extrusion parameters of 3 cm^2^/10 kg. Similarly, approximately 1 ml normal saline was injected via the tail veins of rats in the TP + hUC-MSCs group, and a TP model was established for this group. hUC-MSCs (1 × 10^5^/100 g) were injected via the tail veins of rats in the TP + hUC-MSCs group, and a TP model was established for this group. hucMSC-Exs (10 μg/100 g) were injected via the tail veins of rats in the TP + hucMSC-Exs group (Fig. 1). All rats were sacrificed after 24 h of modeling, and serum and pancreatic tissue were retained for subsequent experiments.

### PKH26 labeled hucMSC-Exs

According to the manufacturer’s instructions for PKH26 dye (BBcellProbe, China), hucMSC-Exs were mixed and incubated with PKH26 in the dark. Then, the solution was centrifuged and the precipitate was retained. The precipitate was resuspended in PBS and incubated again to remove unbound dyes at 37 °C in a 5% CO_2_ incubator. The colonization of hucMSC-Exs in pancreatic tissue was observed by injecting them into the successfully established rat model of TP through the veins.

### Histopathological changes in pancreas tissues

Each group of pancreatic tissues was subjected to hematoxylin and eosin (HE) staining, and pathomorphology was observed under a digital slice scanner (Pannoramic 250, 3DHISTECH, Hungary). The extent of pancreatic edema, hemorrhage, cell necrosis, and inflammatory cell infiltration was scored by three pathologists who were blinded to the experimental groups, according to the previously reported standard of Schmidt [14]. The average score of 10 high-power fields was taken as the final score for each slice.

### Detection of the expression levels of serum amylase, lipase, and inflammatory cytokines using ELISA

After thawing the serum at room temperature, the levels of amylase, lipase, and inflammatory cytokines (IL-6, TNF) were detected using ELISA according to the instructions of ELISA kits.

### Detection of apoptosis using TUNEL assay

The fixed pancreatic tissue was dehydrated, embedded, and then sliced using a digital slice scanner (Pannoramic 250, 3DHISTECH, Hungary). Then, the pancreatic tissue was repaired by citric acid microwave for 8 min and washed with PBS for thrice for 5 min each. A fluorescent TUNEL incubation solution was prepared in the dark and incubated at 37 ℃ for 1 h. The nuclei were stained with DAPI for 15 min. The above-mentioned samples were examined according to the standard operating procedure (SOP) for pathological examination. Finally, the prepared samples were observed under a microscope.

### Determination of the expression levels of apoptotic proteins using western blotting

The concentrations of protein were measured using a BCA protein assay kit. Equal amounts of denatured proteins were separated via sodium dodecyl sulfate–polyacrylamide gel electrophoresis (SDS-PAGE) and then transferred to a PVDF membrane. The membrane was blocked with 5% non-fat milk in TBS and then incubated with the appropriate primary antibody (Bax 1:5000; Bcl-2 1:2000; Caspase-3 1:2000; and β-tubulin 1:2000) at 4 °C for 12 h, followed by incubation with secondary antibodies for 1 h at room temperature. Finally, the protein bands were placed in a chemiluminescence gel imaging system (5200, Shanghai Tianneng Technology Co., Ltd), and the exposure time was appropriately adjusted according to the intensity of the signal.

### Detection of apoptotic gene expression using quantitative reverse transcription-PCR (RT-qPCR)

Total RNA was isolated and extracted from pancreatic tissue. UV spectrophotometry was used to determine the concentration and purity of the extracted total RNA. RNA was reverse-transcribed to cDNA. RT-qPCR was performed in a real-time fluorescence quantitative PCR system using the EasyTM Mix-SYBR kit. The sequences of β-tubulin used in this study were as follows: forward 5′-GGGAAATCGTGCGTGACATT-3′ and reverse 5′-GCGGCAGTGGCCATCTC-3′. The sequences of Bax used were forward 5′-AGACACCTGAGCTGACCTTGGAG-3′ and reverse 5′-TTCATCGCCAATTCGCCTGAGAC-3′. The sequences of Caspase-3 used were forward 5′-GCGGTATTGAGACAGACAGTGGAAC-3′ and reverse 5′-AACCATGACCCGTCCCTTGAATTTC-3′. The sequences of Bcl-2 used were forward 5′-TAGAGAGCGTCAACAGGGAGATG-3′ and reverse 5′-GTGCAGATGCCGGTTCAGGTAC-3′. Each reaction was performed in triplicate.

### Statistical analysis

All the data were analyzed with SPSS 25.0. Data were presented as mean ± SD and analyzed using an independent-sample t-test. Values with *P* < 0.05 were considered statistically significant.

## Results

### Identification of hUC-MSCs and hucMSC-Exs

**Fig. 2:**
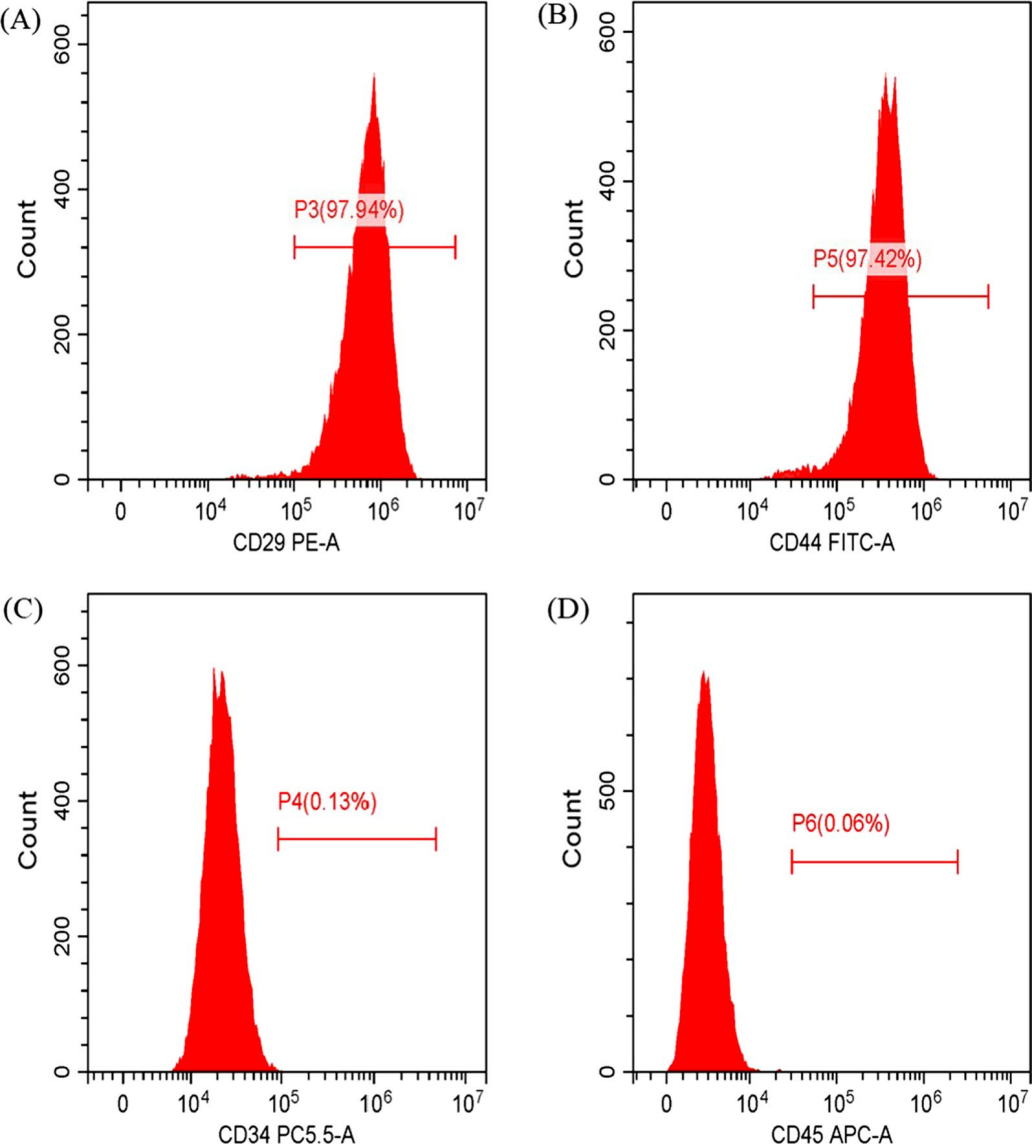
Flow cytometry of hUC-MSCs. Identification of the hUC-MSC surface antigens **a** CD29, **b** CD44, **c** CD34, and **d** CD45

**Fig. 3:**
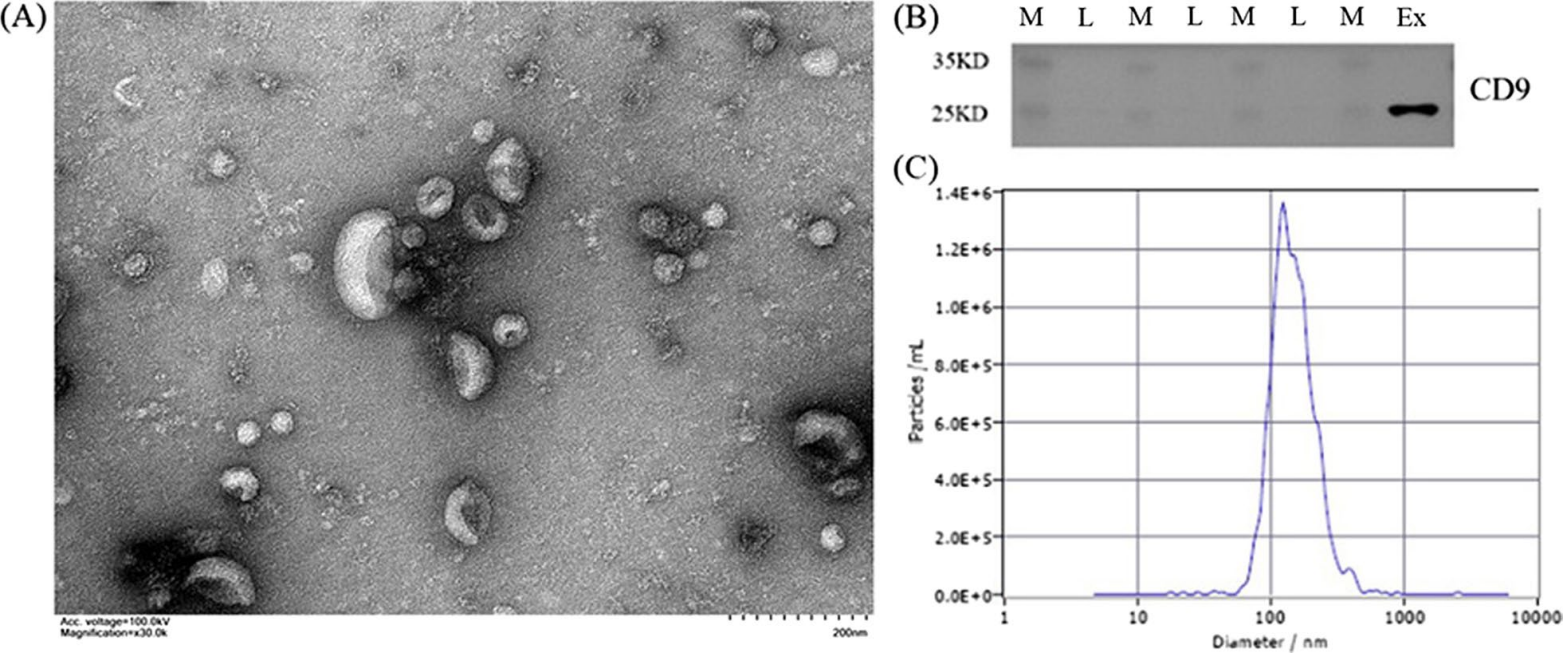
Morphological analysis of hucMSC-Exs. **a** Transmission electron micrograph and **b** particle size and concentration of hucMSC-Exs. **c** Western blot analysis of CD9

To identify hUC-MSCs, flow cytometry was used for detecting the presence of the surface markers CD29, CD44, CD45, and CD34 of umbilical cord mesenchymal stem cells. As shown in Fig. 2, hUC-MSCs were positive for the surface antigens CD29 and CD44 (> 70%) but negative for CD34 and CD45 (< 5%), indicating the successful culture of hUC-MSCs. Using transmission electron microscopy, hucMSC-Exs were identified, and their particle size was determined; using western blotting their concentration was determined. We observed small membranous vesicles with round or oval shape, wherein the periphery of the vesicles was surrounded by membranous structures (Fig. 3). The average particle size and concentration of hucMSC-Exs were 160.2 nm and 7.5 × 10^9^ particles/ml, respectively. The characteristic membrane protein CD9 was detected on of hucMSC-Exs using western blotting.

### In *viv*o colonization of hucMSC-Exs

**Fig. 4:**
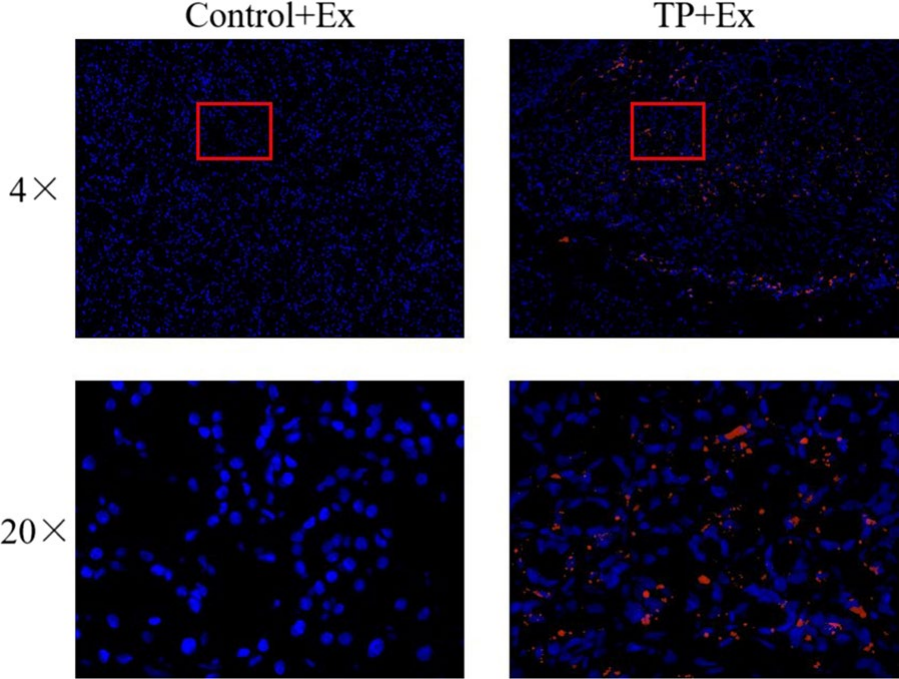
Fluorescence microscopy to assess colonization of hucMSC-Exs in pancreatic tissues. Compared with the Control + Ex group, the TP + Ex group showed evident scattered distribution of red fluorescence

PKH26-labeled hucMSC-Ex was first injected into TP rats via the tail vein, and hucMSC-Ex colonization in injured pancreatic tissues was then assessed using fluorescence microscopy. Figure 4 shows the fluorescence micrographs of pancreatic tissues of the Control + Ex and TP + Ex groups. Compared with the Control + Ex group, the TP + Ex group showed evident scattered distribution of red fluorescence, indicating significant hucMSC-Ex colonization in the TP + Ex group pancreatic tissues. Together, these results suggest that PKH26 can effectively label hucMSC-E and that hucMSC-E tends to be recruited in injured pancreatic tissue.

### Pathological injury to rat pancreas

**Fig. 5:**
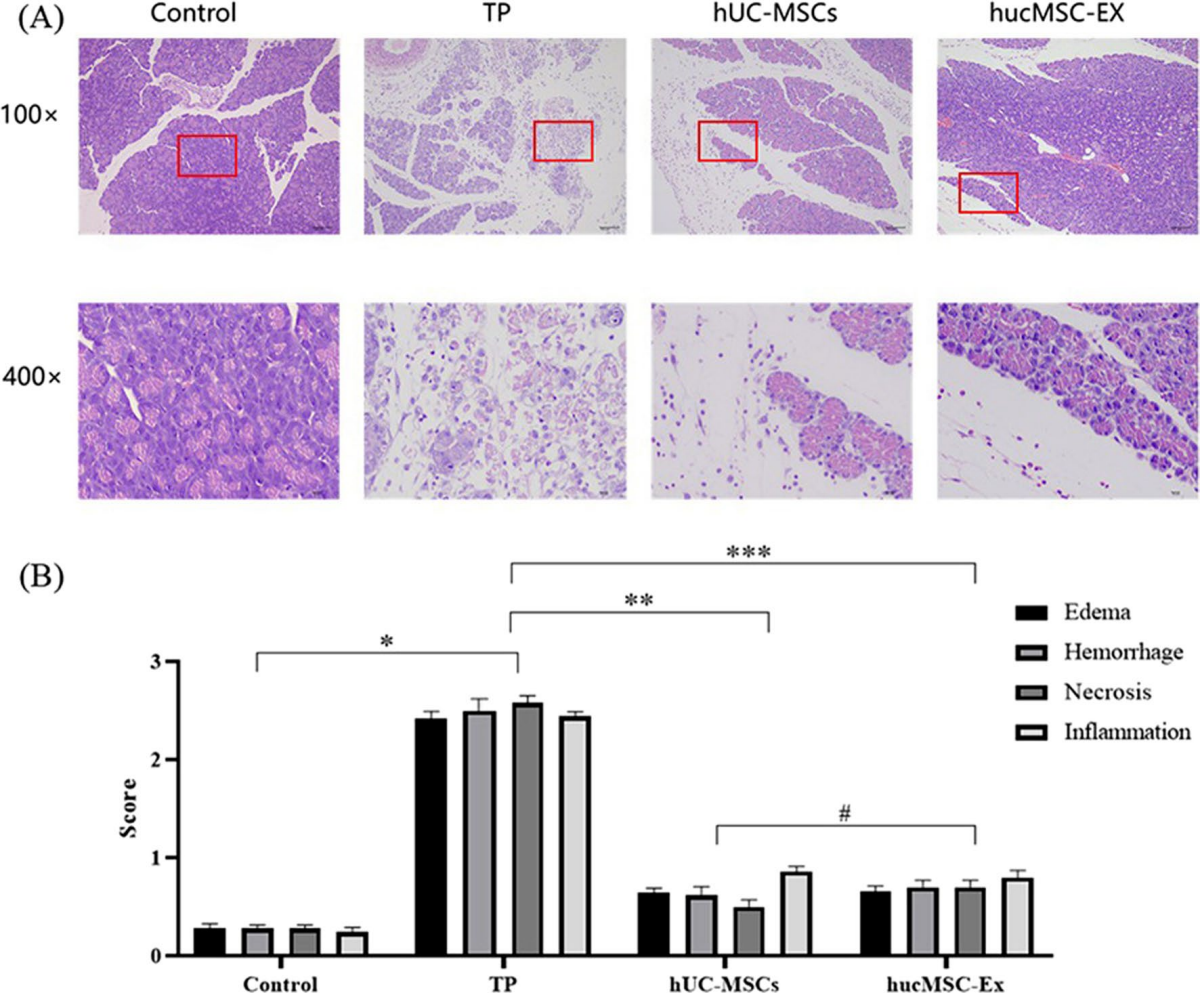
Histopathological evaluation of the pancreas. **a** HE staining of pancreatic tissue. **b** The pancreatic histopathological score. *Control versus TP groups, *P* < 0.05; ** TP versus hUC-MSCs groups, *P* < 0.05; *** TP versus hucMSC-Exs groups, *P* < 0.05; #hUC-MSCs versus hucMSC-Exs groups, *P* > 0.05

The control group pancreatic tissue showed no pathological changes, such as inflammatory cell infiltration, degeneration, and necrosis (Fig. 5a). However, the TP group pancreatic tissue structure was fuzzy and had an unclear boundary, indicating that some acinar cells were necrotic. The necrotic area and stroma were accompanied by inflammatory cell infiltration, and edema was seen in some stromal cells. After the administration of hUC-MSCs and hucMSC-Exs, the islet structure became clear and complete again. Compared with that in the TP group, the degree of pancreatic edema was reduced in the TP + hUC-MSCs and TP + hucMSC-Exs groups, demonstrating significantly reduced infiltration of inflammatory cells in the stroma. In addition, the pathological score of the TP + hUC-MSCs and TP + hucMSC-Exs groups decreased significantly compared with that of the TP group (*P* < 0.05) (Fig. 5b). However, there were no significant differences between the TP + hUC-MSCs and TP + hucMSC-Exs groups (*P* > 0.05).

### Analysis of serum amylase, lipase, and inflammatory cytokines

**Fig. 6:**
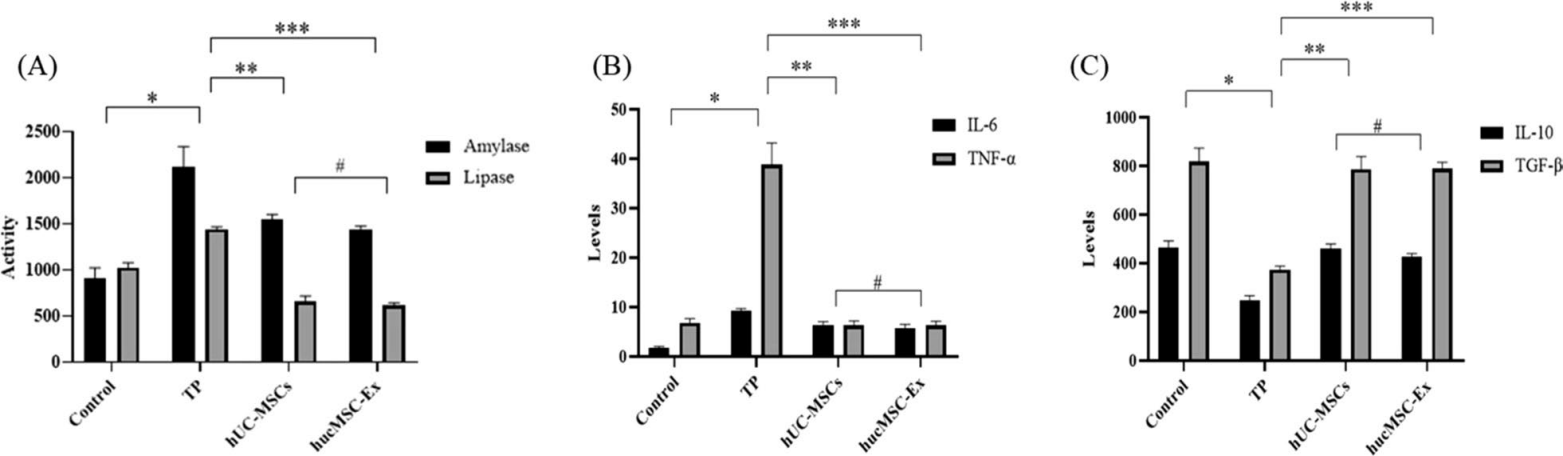
ELISA of the activities of amylase and lipase and levels of inflammatory cytokines. **a** ELISA of amylase and lipase activities. Levels of **b** pro-inflammatory and **c** anti-inflammatory cytokines. *Control versus TP groups, *P* < 0.05; ** TP versus hUC-MSCs groups, *P* < 0.05; *** TP versus hucMSC-Exs groups, *P* < 0.05; #hUC-MSCs versus hucMSC-Exs groups, *P* > 0.05

The activities of serum amylase and lipase in the TP group were higher than those in the control group 24 h after modeling (*P* < 0.05). Compared with that in the TP group, serum amylase and lipase levels were decreased in the TP + hUC-MSCs and TP + hucMSC-Exs groups 24 h modeling (*P* < 0.05). There were no significant differences between the TP + hUC-MSCs and TP + hucMSC-Exs groups (*P* > 0.05) (Fig. 6a).

The levels of pro and anti-inflammatory cytokines in the four groups are presented in Fig. 6b, c. The levels of the proinflammatory cytokines IL-6 and TNF-α in the TP group was significantly higher than that in the control group, whereas the levels of the anti-inflammatory cytokines IL-10 and TGF-β were lower than that in the control group (*P* < 0.05). Compared with that in the TP group, the levels of IL-6 and TNF-α were significantly decreased and the levels of IL-10 and TGF-β were increased in the TP + hUC-MSCs and TP + hucMSC-Exs groups (*P* < 0.05). There were no significant differences between the TP + hUC-MSCs and TP + hucMSC-Exs groups (*P* > 0.05).

### Pancreatic apoptosis

**Fig. 7:**
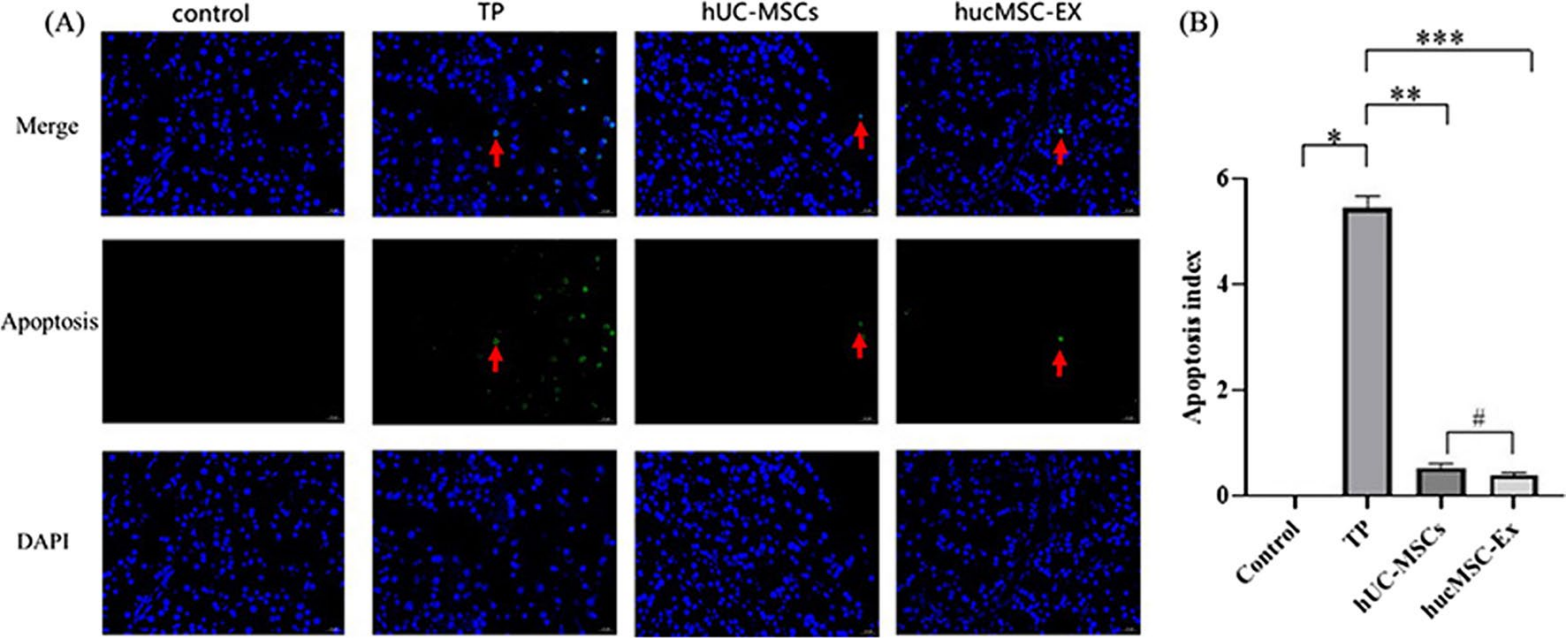
Analysis of pancreatic apoptosis. **a** Apoptosis of pancreatic acinar cells observed using fluorescence microscopy of TUNEL-stained cells; **b** apoptotic index. *Control versus TP, *P* < 0.05; ** TP versus hUC-MSCs, *P* < 0.05; *** TP versus hucMSC-Exs, *P* < 0.05; #hUC-MSCs versus hucMSC-Exs, *P* > 0.05

Pancreatic acinar cells of the TP group underwent apoptosis (Fig. 7a), but not of the control group. Scattered apoptotic cells were observed in the TP group, and the number of apoptotic cells was significantly reduced in the TP + hUC-MSCs and TP + hucMSC-Exs groups.

The apoptotic index of the TP group was significantly higher than that of the control group (*P* < 0.05; Fig. 7b). The TP + hUC-MSCs and TP + hucMSC-Exs groups exhibited significantly lower indices than the TP group (*P* < 0.05). There were no significant differences between the TP + hUC-MSCs and TP + hucMSC-Exs groups (*P* > 0.05).

**Fig. 8:**
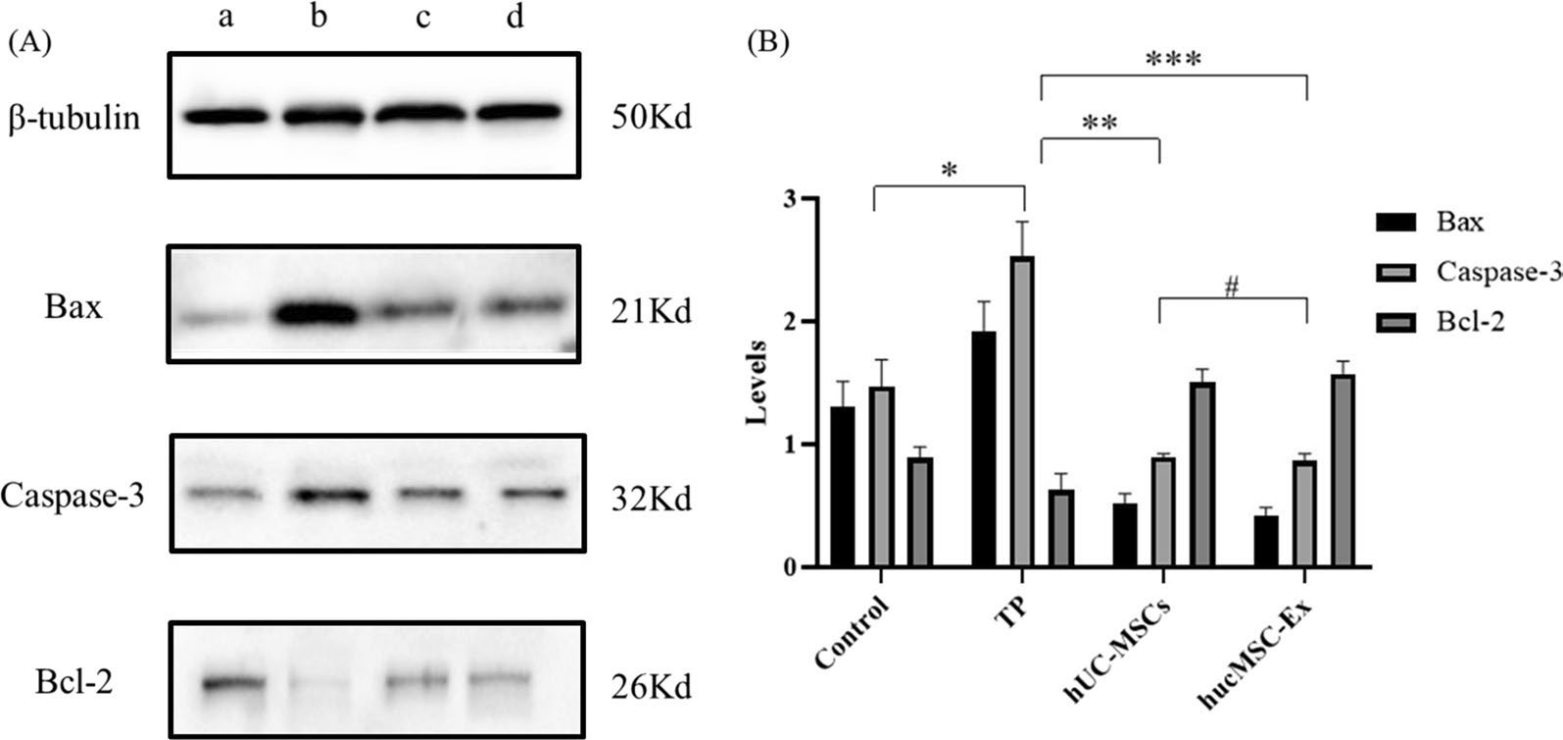
Western blot analysis of apoptotic proteins. **A** The bands of apoptotic proteins. a: Control group; b: TP group; c: hUC-MSCs group; and d: hucMSC-Exs group. **B** The expression of apoptotic proteins. *Control versus TP, *P* < 0.05; ** TP versus hUC-MSCs, *P* < 0.05; *** TP versus hucMSC-Exs, *P* < 0.0; #hUC-MSCs versus hucMSC-Exs, *P* > 0.05

Compared with that in the TP group, the expression levels of the apoptotic proteins Bax and caspase-3 were significantly decreased in the TP + hUC-MSCs and TP + hucMSC-Exs groups. By contrast, the expression levels of the apoptotic protein Bcl-2 was increased in the TP + hUC-MSCs and TP + hucMSC-Exs groups (*P* < 0.05). There were no significant differences between the TP + hUC-MSCs and TP + hucMSC-Exs groups (*P* > 0.05; Fig. 8).

**Fig. 9:**
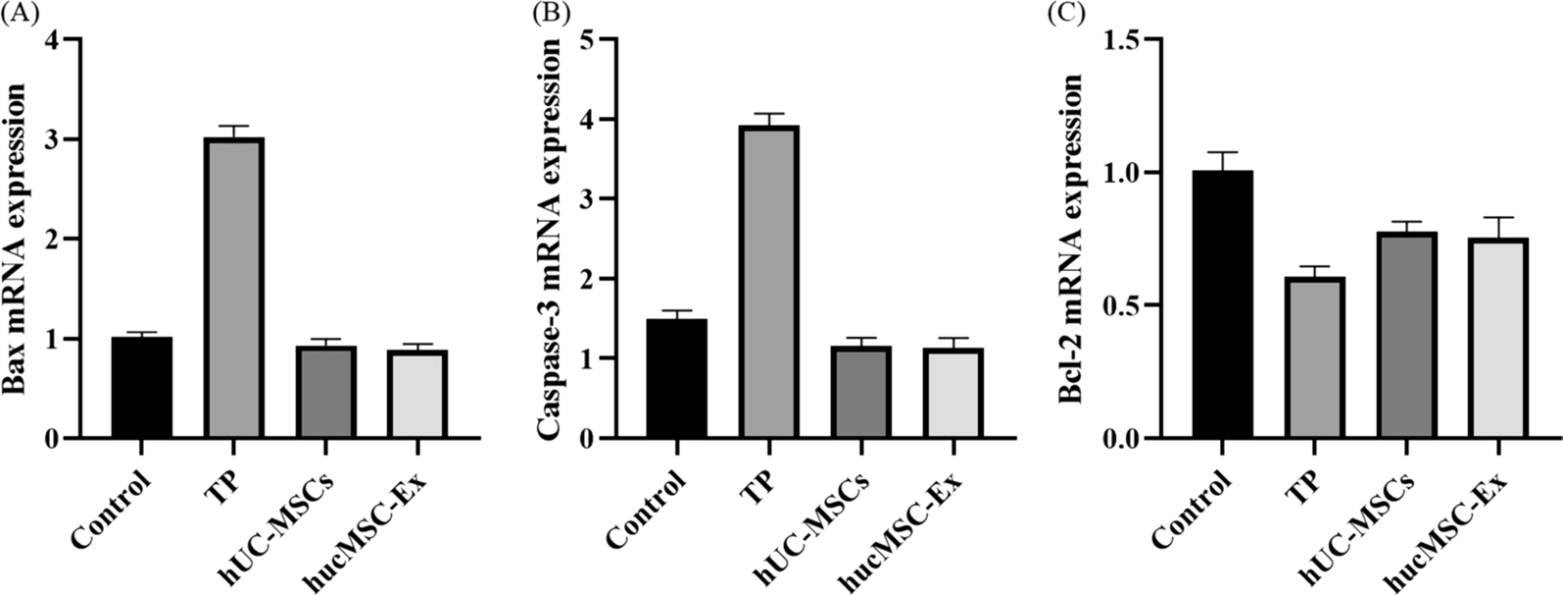
RT-qPCR analysis of mRNA expression levels. **a** Bax; **b** Caspase-3, and (C) Bcl-2 mRNA levels. *Control versus TP, *P* < 0.05; ** TP versus hUC-MSCs, *P* < 0.05; *** TP versus hucMSC-Exs, *P* < 0.05; #hUC-MSCs versus hucMSC-Exs, *P* > 0.05

RT-qPCR results showed that compared with that in the TP group, the expression of Bax and Caspase-3 mRNA in the TP + hUC-MSCs and TP + hucMSC-Exs groups decreased (*P* < 0.05). By contrast, the expression levels of Bcl-2 mRNA were increased in the TP + hUC-MSCs and TP + hucMSC-Exs groups (*P* < 0.05). There was no significant difference between the TP + hUC-MSCs and TP + hucMSC-Exs groups (*P* > 0.05; Fig. 9).

## Discussion

With the advancements in regenerative medicine and tissue engineering techniques, hUC-MSCs with their unique immunomodulatory abilities have drawn considerable attention and been widely used in the fields of tissue repair, regeneration, and recovery of tissue functions. A research study established a rat model of severe acute pancreatitis (SAP) by injecting sodium taurocholate in the pancreatic duct. Subsequently, hUC-MSCs were injected via the tail vein of rats, and the results showed that the transplantation of hUC-MSCs effectively reduced levels of serum amylase and TNF-α as well as decreased the pancreatic dry weight [15]. Another study suggested that hUC-MSCs not only inhibited the secretion of inflammatory cytokines and the activation of pancreatic stellate cells, but also significantly suppressed acinar cell apoptosis via downregulating PI3K signing pathway in pancreatic tissue obtained from a rat model of chronic pancreatitis [16]. Several studies have also revealed that hUC-MSCs may exert their therapeutic effects via paracrine mechanisms that act at the cellular and molecular levels [17–19]. HucMSC-Exs have several advantages such as they are easy to extract, store, and transport; they have low immunogenicity; and they have better biocompatibility. Significantly, hucMSC-Exs have been reported to exert protective effects in diseases of liver and lungs, kidney injury, as well as in diabetes [20–22]. A study showed that hucMSC-Exs improved glomerular atrophy by reducing blood urea nitrogen (BUN), serum creatinine (SCR), and urinary albumin levels, and reduced inflammation and oxidative stress by ameliorating systemic inflammation to repair renal injury and improve renal function [23]. Another study reported that hucMSC-Exs decreased alveolar damage and inflammatory response, and increased the proportion of M2 macrophages in the mice model of systemic lupus erythematosus [24].

In TP, some pancreatic tissue becomes necrotic and dysfunctional. In case of a ruptured pancreatic duct, a large volume of pancreatic juice may spill in surrounding area. Digestive enzymes present in pancreatic juice can further digest pancreatic tissue thereby aggravating pancreatic tissue necrosis. During TP, the segmental or diffuse inflammation of the pancreatic tissues can lead to pancreatic necrosis, fibrosis, and atrophy, and eventually lead to the disappearance of acinar cells and islet cells. This significantly damages pancreatic structure, and severely affects endocrine and exocrine function of pancreas [25, 26]. Therefore, controlling acinar cell apoptosis and improving pancreatic microcirculation is expected to delay post-traumatic necrosis and promote the repair of pancreatic tissue. Exosomes can regulate apoptosis by participating in multiple signaling pathways, and exert powerful immunomodulatory effects by modulating the status of immune cells via immunoactive molecules present in their cargo. In addition, exosomes can upregulate the expression of angiogenesis-related molecules such as FGF-1, VEGFA, and VEGFR-2 to exert pro-angiogenic effects [27–29].

In this study, we established a rat model of TP that simulates the pathophysiological changes of pancreatic tissue after it experiences trauma. This is consistent with the results of previous studies [13]. After generating the model, we injected hUC-MSCs and hucMSC-Exs in rats via their tail veins. The colonization of exosomes was observed in pancreatic tissue. The pancreatic edema was significantly decreased in injured pancreatic tissue of the TP group; the outline of pancreatic tissue was maintained in some of the afflicted areas; the structure of pancreatic lobules was not completely destroyed; normal exocrine cell structure was observed in the injured area; and the infiltration of the inflammatory cells was significantly decreased. Furthermore, compared with TP group, the levels of proinflammatory cytokines TNF-α and IL-6 were significantly decreased in TP + hUC-MSCs group and TP + hucMSC-Exs group, whereas the levels of the anti-inflammatory cytokines IL-10 and TGF-β were significantly increased. This suggested that hUC-MSCs and hucMSC-Exs can exert reparative effects in rats with TP by regulating the expression of inflammatory and anti-inflammatory cytokines. In addition, compared with TP group, the expression levels of Bax and caspase-3 were downregulated in TP + hUC-MSCs and TP + hucMSC-Exs groups; the expression of Bcl-2 was upregulated in TP + hUC-MSCs and TP + hucMSC-Exs groups. Similarly, TUNEL staining results also suggested that hUC-MSCs and hucMSC-Exs can repair TP tissue by inhibiting apoptosis of pancreatic cells. Based on our results, we conclude that in rats, hucMSC-Exs can exert comparable therapeutic effects as hUC-MSCs for TP. However, hucMSC-Exs have the advantage of being smaller in size, less complex, and easier to produce and store than their parent cells. Furthermore, hucMSC-Exs are also less immunogenic than their parent cells owing to their lower content of membrane-bound proteins. Therefore, hucMSC-Exs are potential candidates for cell-free "stem cell"-based alternative treatment in patients with TP and needs to be evaluated further in the future studies.

## Conclusion

HucMSC-Exs can attenuate pancreatic tissue injury and accelerate pancreatic repair in rat model of TP. This is realized via inhibiting acinar cell apoptosis and controlling systemic inflammatory responses. However, the specific molecular mechanisms underlying these therapeutic effects of hucMSC-Exs remain unknown. Thus, extensive in-depth studies are needed in the future before their application in clinical settings.

## Data Availability

Availability of data and materials were available in the manuscript.

## Abbreviations

TP: Traumatic pancreatitis
AP: Acute pancreatitis
SAP: Severe acute pancreatitis
hUC-MSCs: Human umbilical cord mesenchymal stem cells
hucMSC-Exs: Human umbilical cord mesenchymal stem cells-exosomes
SD: Sprague–dawley
IL: Interleukin
TNF: Tumor necrosis factor
SDS-PAGE: Sodium dodecyl sulfate–polyacrylamide gel electrophoresis
PVDF: Polyvinylidene difluoride
TBS: Tris-buffered saline
BUN: Blood urea nitrogen
SCR: Serum creatinine
TGF: Transforming growth factor
